# Multimorbidity resilience and health behaviors among older adults: A longitudinal study using the Canadian Longitudinal Study on Aging

**DOI:** 10.3389/fpubh.2022.896312

**Published:** 2022-09-23

**Authors:** Andrew Wister, Lun Li, Carly Whitmore, Jennifer Ferris, Katarzyna Klasa, Igor Linkov

**Affiliations:** ^1^Department of Gerontology, Gerontology Research Centre, Simon Fraser University, Vancouver, BC, Canada; ^2^School of Social Work, MacEwan University, Edmonton, AB, Canada; ^3^School of Nursing, McMaster University, Hamilton, ON, Canada; ^4^Gerontology Research Centre, Simon Fraser University, Vancouver, BC, Canada; ^5^BC Observatory for Population and Public Health, BC Centre for Disease Control, Vancouver, BC, Canada; ^6^University of Michigan School of Public Health, Ann Arbor, MI, United States; ^7^United States Army Corps of Engineers, Engineering Research and Development Center, Vicksburg, MS, United States; ^8^Carnegie Mellon University, Pittsburg, PA, United States

**Keywords:** multimorbidity, resilience, aging, health behaviors, CLSA

## Abstract

**Objective:**

There has been a growing interest in examining why some individuals adapt and bounce back from multimorbidity (resilience) better than others. This paper investigates the positive role of protective health behaviors on multimorbidity resilience (MR) among older adults focusing on older persons with two or more concurrent chronic conditions, and separately for three multimorbidity chronic illness clusters.

**Methods:**

Using Baseline and Follow-up One data from the Comprehensive Cohort of the Canadian Longitudinal Study on Aging, we studied 10,628 participants aged 65 years and older who reported two or more of 27 chronic conditions, and three multimorbidity clusters: Cardiovascular/metabolic, Musculoskeletal, and Mental health. Associations between health behaviors and MR were evaluated using Linear Mixed Models, adjusting for socio-demographic, social/environmental, and illness context social determinants of health.

**Results:**

Among older adults with two or more illnesses, smoking, satisfaction with sleep, appetite, and skipping meals were associated with MR in the expected direction. Also, obesity (compared to normal weight) and skipping meals showed longitudinal interaction effects with survey wave. Most of the results were replicated for the physical multimorbidity clusters (Cardiovascular/metabolic and Musculoskeletal) compared to the full 2+ multimorbidity analyses; however, for the Mental health cluster, only satisfaction with sleep was supported as a lifestyle predictor of MR.

**Discussion:**

Several modifiable health behaviors identified in the broader health and aging literature are important in affecting levels of multimorbidity resilience in older age. These factors are important strength-based areas to target. Additionally, several social determinants of health are also supported and parallel research on multimorbidity risk. The effects of lifestyle factors for resilience among older adults is dependent on the type of multimorbidity measured. We conclude that the results have significant public health, program intervention, and clinical implications for healthy aging among persons coping with multimorbidity.

## Introduction

In response to the dominant pathogenic approach to understanding health, researchers have begun to examine salutogenic responses to illness-related adversities by which individuals maintain and regain a sense of wellness in their lives through positive adaptation processes (1). There has also been a recognition that the majority of individuals experience illness adversity in older age, and that definitions of successful aging have not recognized the strength-based responses among individuals who may have been considered as “not aging well” (2, 3). This has led to interest in understanding one's ability and resources needed to cope with and navigate stress-inducing experiences—termed *resilience* (4, 5). Resilience models have been applied to numerous forms of adversity across the lifecourse, including disasters, aging-related functional loss, mental illness and multimorbidity (6–16), the latter of which is the focus of this paper (17).

In most economically advanced countries, approximately two-thirds of older adults have multimorbidity (two or more concurrent chronic conditions) and these rates increase with advanced age (18, 19). Living with multimorbidity represents a particularly unique and potentially potent form of adversity, since it can compound the synergetic deleterious effects of individual chronic conditions that shape symptom burden, functional ability, quality of life, and result in higher health care costs (20–24). Thus, a robust body of research has arisen addressing forms of multimorbidity from a resilience perspective (15, 19, 25, 26). The broad set of adaptations, including partial recovery and potentially reintegration (functional roles, identity, etc.), have been termed *multimorbidity resilience* (MR) (15, 25). However, there remains a gap in research knowledge pertaining to predictors of multimorbidity resilience, in particular, longitudinal analyses of health behaviors that have significant public health implications. These are important since they are mutable predictors of coping and adaptation to multimorbidity. Our primary research question is: what are the modifiable behavioral protective/risk factors that are associated with multimorbidity resilience over time?

## Conceptualizing multimorbidity resilience

The science of resilience has identified a number of levels within which processes of adaptation and recovery can be manifested through interaction across psychological, emotional, spiritual, physical/functional, economic, cultural, and complex system domains (6, 16, 27–29). Specifically, in gerontology, the availability and accessibility of resources and the ability for older individuals to harness them constitute resilience and can shape multimorbidity trajectories. Yet some individuals are more likely to possess various protective factors, such as healthy lifestyle routines, social support systems, economic resources, and social-psychological strengths that may enable them to cope better than others with multimorbidity deficits (25, 30).

Given the focus of this research, we utilize the Lifecourse Model of Multimorbidity Resilience (LMMR) to frame the analyses [for full description, see (31)]. The LMMR uses three primary resilience domains: (A) *Functional resilience* which is necessary for completing tasks of daily living, social roles, and remaining physically active (27, 32). (B) *Social resilience* for the maintenance of positive social and community connectedness (10, 25, 33), as well as protection against feelings of loneliness and experiences of social isolation (34–36). (C) *Psychological resilience* which is needed to mentally cope with stressors linked to multimorbidity, rooted in stress theory and the cognitive appraisal process (37). The LMMR forms the basis for framing the analyses and developing the measurement strategy (see Section Methods).

## Health behaviors and multimorbidity resilience

Research into precursors of multimorbidity and associated morbidity and mortality outcomes has identified a number of important modifiable health behaviors that we expect to predict MR (38, 39). Evidence has established that smoking, physical activity, obesity, eating habits, nutrition and alcohol consumption are associated with multimorbidity, although findings are equivocal for some of these behaviors (30, 38–42). In a cohort analysis, Canizares et al. (42) showed that there were successive cohort increases in multimorbidity inflated by being obese, a smoker, and engaging in a sedentary lifestyle. Other studies found associations between smoking and multimorbidity (43, 44); and poor eating habits and obesity and multimorbidity (40). However, studies have uncovered inconsistent findings for physical activity (41, 42, 45), and alcohol consumption (39). Additionally, while sparse, research has shown a positive influence of sleep patterns for recovery (46), and that individuals with sleep disturbances progress to multimorbidity more rapidly over the life span (47). Health behaviors assist in the management and adaptation to illness-related stressors, foster stronger social connections and support, and enhance wellbeing (48–51). We hypothesize that the above health behaviors will be positively (protective factors) or negatively (risk factors) associated with levels of MR in older adults.

There are a number of other social determinants of multimorbidity that need to be adjusted to test the above hypotheses, including age, sex, education, income, marital status, immigration status, social support, housing and urban/rural residence (25, 30, 33, 38, 39, 42, 52). Additionally, perceptions of pain, medication use, and perceived health are indicators of the type and severity of multimorbidity that also should be included as covariates (25). This is because perception of pain is expected to have an inverse association with MR, due to its debilitating effect on functioning (26), while perceived health has been anticipated to be positively related to MR (9, 21, 53). Finally, medication use is associated with severity of illness but may also mitigate illness symptoms (26).

## Methods

### Data and sample

Participants were drawn from the Baseline and Follow-up 1 (FUP1) data of the Comprehensive cohort of the Canadian Longitudinal Study on Aging (CLSA). The CLSA is a national-level population-based longitudinal survey collecting social, psychological, biological, and clinical data from 51,338 Canadians aged 45–85 years old when recruited starting in 2011. Currently, two waves of CLSA data are available: Baseline data on 51,338 participants (2011 to 2015), and Follow-up One (FUP1) with 44,817 participants (2015 to 2018), separated by ~3 years. The CLSA is comprised of two cohorts of participants, the Comprehensive cohort who were randomly selected among population residing within 25 km (or 50 km in a lower population density area) of the 11 data collection sites across Canada, and the Tracking cohort who were randomly selected from the ten provinces by the computer-assisted interview system. Detailed information about the CLSA has been published elsewhere (54–56). Researchers can access the de-identified data, and information on weighting through the CLSA website (www.clsa-elcv.ca).

**Figure 1:**
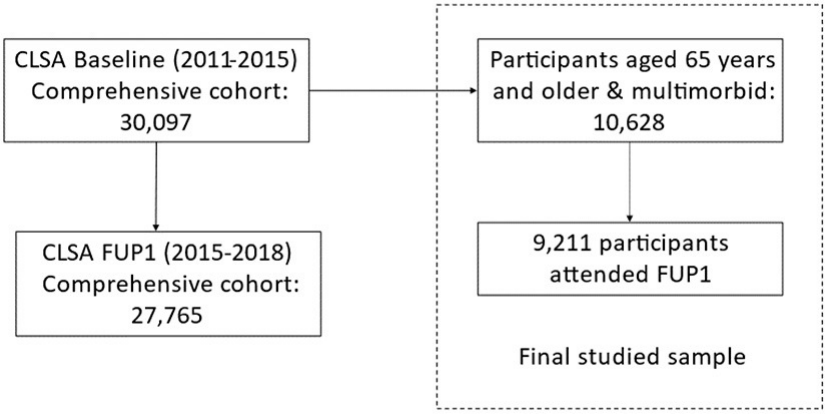
Flow chart of CLSA sample selection.

The current study was conducted based on the Comprehensive cohort only, since several physiological measures essential to the multimorbidity resilience index measure (see below) are only available in this cohort. There are 30,097 comprehensive participants at Baseline of whom 27,765 (92.3%) are included in FUP1. There were 10,628 participants aged 65 years and older with two or more chronic health conditions at the Baseline included in the present analyses (refer to Figure 1 for the process of sample selection). During data collection, participants were asked whether they have been told by a doctor that they had the following 27 types of chronic conditions, including Alzheimer's disease, back problems, bowel incontinence, cancer, cataracts, diabetes, epilepsy, glaucoma, heart attack, heart disease, high blood pressure, irritable bowel syndrome, kidney disease, Parkinson's disease, peripheral vascular disease, lung disease, macular degeneration, multiple sclerosis, osteoarthritis, osteoporosis, migraine headaches, rheumatoid arthritis, stroke, thyroid problem, transient ischemic attack, ulcer, and urinary incontinence. In our study, participants diagnosed with two or more chronic illnesses were deemed multimorbid.

Additional sub-sample analyses were conducted based on sub-sets participants (65+) with two or more conditions within three exclusive multimorbidity clusters: (1) Cardiovascular/Metabolic cluster (heart disease, diabetes, and high blood pressure) (3,033), (2) Musculoskeletal cluster (osteoarthritis, osteoporosis, and lower back problem) (2,417), and (3) Mental health cluster (mood disorder, anxiety disorder, and migraine headache) (753). The three chronic illnesses within each cluster have been found to occur concurrently and share similar symptoms (57–62). These grouping were based on review of the cluster literature and the likely co-occurrence of particular chronic illness groupings.

### Measurement

#### Dependent variable

The multimorbidity resilience index (MRI) is the dependent variable in this study. The MRI was developed by Wister et al. (15) based on the LMMR using CLSA Comprehensive cohort Baseline data. The MRI contains three resilience domains essential to aging-related adversity and adaptation, including functional, social, and psychological multimorbidity resilience domains. In each resilience domain, three adversity challenges or positive adaptation variables were selected to calculate the sub-index score. The functional resilience domain includes the Summary Performance Score of Functional Ability Scale (63), the Older Americans Resources and Services Activities of Daily Living (ADL) Scale, and Instrumental Activities of Daily Living (IADL) Scale (64). The social resilience domain contains the total Medical Outcomes Study (MOS) Social Support Survey (65), a social participation measure related to the frequency of participation in activities with family and friends, and a single item measuring perceived loneliness over the past week (from the Center for Epidemiological Studies Depression - CES-D scale). The psychological resilience domain variables are the (CES-D) 9 item Scale (66) with the loneliness item removed, the Kessler Psychological Distress K10 Scale (67), and the Diener Satisfaction with Life Scale (68). The removal of the loneliness item from the CES-D has been used in research on loneliness, depression, and aging using the revised CES-D scale (69). This research demonstrated that the removal of a single item in the CES-D scale had a minimal effect, although comparisons made to the full 10 item CES-D should be made with caution.

A mapping system was applied to standardize different measurement types and skewed distributions of measures by converting all of the index related variables into a score between 0 and 10. For a full description of this index and the mapping system, see (15). This mapping system with normalization procedure has been used to develop other validated indices, such as a frailty index (70). The total MRI score was calculated by adding the three sub-index scores and dividing by three to produce a measure with the same range (0–10). Higher MRI scores mean greater multimorbidity resilience. The initial analyses have established good concurrent validity of the MRI (15). MRI total scores were associated with perceived health (OR = 1.68, CI 1.59–1.77); sleep quality (OR = 1.34, CI 1.30–1.38); perceived pain (OR = 0.80, CI 0.77–0.83); hospital overnight stays (OR = 0.87, CI 0.83–0.91); and emergency department visits (OR = 0.90, CI 0.87–0.94), after adjusting for socio-demographic factors, and number of chronic conditions. In the present study, at Baseline, the MRI mean was 6.47 (standard deviation = 1.64).

#### Socio-demographic variables

Six socio-demographic covariates based on the CLSA Baseline data included: age, gender, education, household income, marital status, and immigration status. Participants' age was measured in single years and ranged from 65 to 86. Sex was measured as “male” and “female.” The highest educational attainment was coded at four levels from “no post-secondary education”, “trade certificate or diploma or equivalent,” “bachelor's degree,” to “university degree above bachelor's degree.” The annual household income was categorized into five groups, including “<$20,000,” “$20,000–49,999,” “$50,000–99,999,” “$100,000–149,000,” and “$150,000 and over.” Marital status was originally collected based on five categories, and further recoded into two groups as “not married” (single, never married, widowed, divorced, separated) and “married/common-law.” Immigration status was based on participants' country of birth and grouped into “immigrants” and “born in Canada.”

#### Social and environmental variables

Four social and environmental covariates were included: number of friends, number of relatives, housing problems, and residential area. In the CLSA, participants were asked about the number of people they considered as close friends with whom they shared personal matters (ranging from 0 to 90), and the number of living relatives (ranging from 0 to 100). For those participants who reported at least one of the seven housing-related problems (noise, leaking, condensation, electrical wiring or plumbing, heating, maintenance or repairs, and infestations) were grouped into “with housing problem(s),” and others into “no housing problem.” Residential areas were coded dichotomously as “rural area” and “urban area.”

#### Behavioral and lifestyle variables

Six primary health behavior variables were of interest for these analyses, including physical inactivity, alcohol consumption, smoking, sleep, appetite, and skipped meals. Physical inactivity was measured using a measure of sedentary behavior from the Physical Activity Scale for the Elderly (PASE) (71). Participants were asked about the daily amount of time for sitting activities, ranging from “<30 min,” “30 min but <1 h,” “1 h but <2 h,” “2 h but <4 h,” and “4 h or more.” Alcohol consumption is an aggregated variable based on a series of variables capturing participants' consumption (by drinks) of beer, wine, liquor, and other types of alcohol during both weekdays and weekends. The National Institute on Alcohol Abuse and Alcoholism (72) guidelines were used to group the variable at two levels: “14 or less drinks per week” and “15 or more drinks per week,” since this cut-off reflects potentially problematic alcohol consumption. Smoking was measured based on participants' smoking activities during the past 30 days before taking the survey. A dichotomous variable was created as “smoked” and “not smoking in the last 30 days.” Participants evaluated their sleeping quality at five levels: “very dissatisfied,” “dissatisfied,” “neutral,” “satisfied,” and “very satisfied.” Appetite was similarly self-reported and measured as “poor,” “fair,” “good,” and “very good.” An additional variable was available capturing frequency of skipped meals. This variable was recoded as: “all the time to sometimes,” and “rarely or never.” Both the Baseline and FUP1 data of these variables were used in the data analysis.

#### Health context variables

Four main health related variables were examined, including Body Mass Index (BMI), self-rated health, pain, and number of medications. BMI was categorized into four levels: “underweight” (18.49 or below), “normal” (18.5–24.9), “overweight” (25–29.9), to “obese” (30 or higher). Self-rated health was measured using a single ordinal scale categorized as “poor,” “fair,” “good,” “very good,” and “excellent.” Pain was measured based on responses to the usual intensity of pain or discomfort: “none,” “mild,” “moderate,” and “severe.” The number of medications variable is an aggregated variable based on ten questions related to medication taken or not for ten highly prevalent chronic conditions, including arthritis, diabetes, hypertension, ischemic heart disease, osteoporosis, Parkinson's disease, respiratory problems, stroke, thyroid, and transient ischemic attack. Responses to these questions were combined into a continuous variable with possible scores ranging from 0 to 10 with 10 reflective of a higher number of medications taken. The data from both Baseline and FUP1 were included for the self-rated health and pain variables, but only Baseline data were available for the number of medications.

### Data analytic procedure

Data analyses were performed using SPSS version 26. The descriptive statistics for all variables at both Baseline and FUP1 waves are illustrated in Table 1. Linear Mixed Models [LMM; (73)] were applied to the longitudinal analysis of MRI from Baseline to FUP1 among aging participants with multimorbidity, as well as among the three multimorbidity clusters. LMM has been widely used to analyze panel data as it can control for random effects due to repeated measures on the same participant, and account for within-subject and between-subject variability (74). Additionally, use of LMM allows for both time-invariant (e.g., sex) and time-variant (e.g., age, number of chronic conditions) factors to be included in the same model.

**Table 1:** Socio-demographic, social and environmental, behavioral and lifestyle, and health contextual information among participants.

**Variables**	**Baseline**	**Follow-up 1**	**χ ^2^(df)/*t*-test (df)**
**Age**	73.17 (5.67)	–	
**Gender**			
Male	43.68	–	
Female	56.32		
**Education level**			
No post-secondary education	10.60	–	
Trade certificate or diploma or equivalent	39.96		
Bachelor's degree	23.93		
University degree above bachelor's degree	25.51		
**Household income**			
<$20,000 per year	7.70	–	
$20,000–49,999 per year	35.71		
$50,000–99,999 per year	38.40		
$100,000–149,000 per year	12.26		
$150,000 and over per year	5.92		
**Marital status**			
Not Married	34.57	–	
Married/Common law	65.43		
**Immigration status**			
Immigrants	21.71		
Born in Canada	78.29		
**Number of friends**	5.25 (6.39)	5.51 (7.07)	−1.08
**Number of relatives**	29.96 (25.77)	28.37 (24.26)	4.19^***^
**Housing problems**			
Yes	18.11	19.21	380.02 (1)^***^
No	81.89	80.79	
**Urban, rural status**			
Rural area	8.68	6.74	2,439.89 (1)^***^
Urban area	91.32	93.26	
**BMI**			
Underweight	0.89	0.97	9,046.38 (9)^***^
Overweight	41.74	39.02	
Obese	29.42	31.78	
Normal	27.95	28.23	
**Physical inactivity**			
1 h to <2 h	10.68	6.81	587.98 (9)^***^
2 h to <4 h	41.97	33.50	
4 h and more	45.52	58.50	
Siting <1 h	1.82	1.19	
**Alcohol consumption**			
14 drinks or less per week	92.50	93.44	1,620.64 (1)^***^
15 drinks or more per week	7.50	6.56	
**Smoking**			
Not in the last 30 days	94.87	96.10	4,130.26 (1)^***^
Smoked	5.13	3.90	
**Sleep**			
Very satisfied	20.89	22.70	2,422.16 (16)^***^
Satisfied	40.89	37.55	
Neutral	14.11	16.32	
Dissatisfied	19.93	18.05	
Very dissatisfied	4.18	5.39	
**Appetite**			
Very good	50.54	12.37	2,483.89 (9)^***^
Good	40.95	20.23	
Fair	6.38	5.07	
Poor	2.13	62.33	
**Skipped meals**			
Rarely or never	19.08	23.32	1,470.10 (1)^***^
All the time to sometimes	80.92	76.68	
**Self-rated health**			
Excellent	16.44	14.79	3,254.94 (16)^***^
Very good	39.05	37.99	
Good	33.79	33.60	
Fair	9.12	11.08	
Poor	1.60	2.54	
**Pain**			
Mild	15.78	12.37	1,652.51 (9)^***^
Moderate	22.98	20.23	
Severe	5.21	5.07	
None	56.03	62.33	
**Number of medications^+^**	1.46 (1.14)	–	
**Multimorbidity resilience index**	6.47 (1.64)	6.22 (1.82)	7.86^***^

^*^p < 0.05; ^**^p < 0.01;

*** *p* < 0.001.

+ This variable is only available at the Baseline wave.

Four hierarchical models representing the four blocks of variables (socio-demographic factors, social and environmental factors, behavioral and lifestyle factors, and the health context factors) were added sequentially into the models. In order to capture the change of MRI from Baseline to FUP1, survey wave analysis was used to test for time-related interaction effects. A random intercept was included to model the variation in the dependent variable outcomes across participants. Likelihood ratio tests based on the Akaike Information Criterion (AIC) were performed to compare the model fit, where a lower value of AIC indicates a better model fit. As recommended by the CLSA methods group (https://www.clsa-elcv.ca/), the trimmed weights were applied for descriptive analysis, and the analytic weights were applied for bivariate and multivariate analyses. We used the LMM function to handle the missing data for different waves on the outcome variables *via* restricted maximum likelihood estimation; and listwise deletion was used for independent variables (e.g., demographic factors) with missing cases. The only exception is the household income variable, which contains 6.4% missing values (over the 5% threshold), and therefore, missing cases were replaced with “not stated.”

## Results

A total of 10,628 participants aged 65 years and older reported two or more chronic conditions at Baseline. The average age was 73. More female older adults were multimorbid than their male counterparts (56 vs. 44%). The majority (89%) of participants reported post-secondary education, with half of them having received university degrees. About three quarters (74%) of participants reported household income between $20,000 and 99,999, and most (65%) were married or living with a partner in common-law relationship. Almost eight in ten (78%) were born in Canada (see Table 1).

The comparative results across survey time periods are also presented in Table 1, and only statistically significant results are described below. Participants reported lower MRI scores at the FUP1 than Baseline (6.22 vs. 6.47, *p* < 0.001). Participants had fewer relatives from Baseline to FUP1 (29.96 vs. 28.37, *p* < 0.001), but no change in the number of close friends. Compared to Baseline, a higher proportion of participants at FUP1 experienced housing problem(s) (19 vs. 18%, *p* < 0.001), and lived in urban area (93 vs. 91%, *p* < 0.001). Also, a higher percentage of participants at FUP1 spent four or more hours in sitting-based activities every day (59 vs. 46%, *p* < 0.001), and rated their appetite as fair to poor (11 vs. 7%, *p* < 0.001) when compared to Baseline. A higher proportion of participants at FUP1 did not smoke in the past 30 days (96 vs. 95%, *p* < 0.001), consumed 14 or less drinks every week (93 vs. 92%, *p* < 0.001), and rarely or never skipped a meal (23 vs. 19%, *p* < 0.001), comparing to Baseline. In addition, the proportion of participants rating their health as poor or fair was higher at FUP1 than Baseline (14 vs. 11%, *p* < 0.001). Satisfaction with sleep decreased, and the pattern of BMI shifted, with a higher proportion in the obese group at FUP1. Finally, a higher proportion of participants reported no pain at the FUP1 than Baseline (62 vs. 56%, *p* < 0.001).

The results of LMM of MRI score among participants with multimorbidity and additional three multimorbidity clusters are presented in Table 2. Only full model in each analysis with all variables included are discussed. As shown in Table 2, participants reported a lower MRI score at FUP1 than Baseline [estimate = −0.77, 95% CI: (−1.42, −0.13)]. Age was significantly related to MRI scores negatively [estimate= −0.03, 95% CI: (−0.04, −0.03)]. Male participants had a significantly higher MRI score than female participants [estimate = 0.34, 95% CI: (0.27, 0.41)]. Only participants with bachelor's degree reported higher MRI scores than that of participants without any post-secondary education [estimate = 0.13, 95% CI: (0.01, 0.25)]. All the income groups above $20,000 per year reported higher MRI scores than participants with an annual household income < $20,000 [estimate= 0.32, 95% CI: (0.19, 0.45) for $20,000–49,999; estimate = 0.49, 95% CI: (0.35, 0.63) for $50,000–99,999; estimate = 0.66, 95% CI: (0.50, 0.83) for $100,000–149,000; estimate = 0.74, 95% CI: (0.55, 0.93) for $150,000 and over]. The MRI scores of unmarried participants were lower than married or partnered participants [estimate = −0.37, 95% CI: (−0.45, −0.30)]. Immigration status was not significantly associated with the MRI.

**Table 2:** Linear mixed models of multimorbidity resilience score in full multimorbidity sample, the cardiovascular/metabolic cluster, the musculoskeletal cluster, and the mental health cluster.

	**Multimorbidity sample**		**Cardiovascular/metabolic cluster**		**Musculoskeletal cluster**		**Mental health cluster**	
	**Estimate**	**95% CI**	**Estimate**	**95% CI**	**Estimate**	**95% CI**	**Estimate**	**95% CI**
**Survey wave**								
Follow-up 1 Baseline (ref.)	−0.77^*^	−1.42, −0.13	−0.75	−1.93, 0.43	−0.97	−2.29, 0.36	−0.59	−1.90, 3.07
**(1) Sociodemographic model**								
**Age**	−0.03^***^	−0.04, −0.03	−0.04^***^	−0.05, −0.03	−0.04^***^	−0.05, −0.02	−0.04^***^	−0.06, −0.02
**Gender**								
Male Female (ref.)	0.34^***^	0.27, 0.41	0.44^***^	0.30, 0.58	0.37^***^	0.20, 0.54	0.25	−0.06, 0.55
**Education level**								
Trade certificate or diploma or equivalent Bachelor's degree University degree above bachelor's degree No post-secondary education (ref.)	0.06 0.13^*^ 0.07	−0.05, 0.17 0.01, 0.25 −0.05, 0.20	−0.14 0.10 −0.10	−0.36, 0.07 −0.14, 0.34 −0.34, 0.15	0.12 −0.02 0.01	−0.12, 0.36 −0.28, 0.24 −0.25, 0.27	0.19 0.05 0.06	−0.23, 0.61 −0.41, 0.51 −0.40, 0.52
**Household income**								
$20,000–49,999 per year $50,000 to $99,999 per year $100,000 to $149,000 per year $150,000 and over per year <$20,000 per year (ref.)	0.32^***^ 0.49^***^ 0.66^***^ 0.74^***^	0.19, 0.45 0.35, 0.63 0.50, 0.83 0.55, 0.93	0.29^*^ 0.53^***^ 0.80^***^ 0.76^***^	0.03, 0.54 0.26, 0.80 0.48, 1.12 0.39, 1.13	0.50^***^ 0.71^***^ 0.83^***^ 0.66^**^	0.24, 0.76 0.43, 0.99 0.48, 1.18 0.25, 1.08	0.63^**^ 0.75^***^ 0.83^**^ 0.39	0.22, 1.03 0.31, 1.20 0.21, 1.44 −0.34, 1.11
**Marital status**								
No married Married/Common law (ref.)	−0.37^***^	−0.45, −0.30	−0.32^***^	−0.48, −0.17	−0.33^***^	−0.48, −0.18	−0.24	−0.52, 0.04
**Immigration status**								
Immigrants Born in Canada (ref.)	−0.04	−0.11, 0.03	0.03	−0.12, 0.18	−0.04	−0.19, 0.12	−0.13	−0.45, 0.19
**(2) Social/environmental model**								
**Number of friends**	0.02^***^	0.01, 0.02	0.01^*^	0.002, 0.02	0.02^***^	0.01, 0.03	0.04^**^	0.01, 0.06
**Number of friends × survey wave**	−0.01^**^	−0.01, −0.002	−0.01	−0.02, 0.01	−0.01	−0.02, 0.01	−0.02	−0.04, 0.01
**Number of relatives**	0.001*	0.001, 0.002	0.002	−0.01, 0.004	0.001	−0.01, 0.003	0.001	−0.003, 0.01
**Number of relatives × survey wave**	0.003^***^	0.002, 0.004	0.005^**^	0.002, 0.008	0.01^***^	0.002, 0.01	0.01	−0.001, 0.01
**Housing problems**								
Yes No (ref.)	−0.31^***^	−0.39, −0.24	−0.29^***^	−0.44, −0.14	−0.31^***^	−0.46, −0.15	−0.34^*^	−0.61, −0.06
**Housing problems × survey wave**								
No × survey wave Yes × survey wave (ref.)	−0.01	−0.11, 0.10	−0.03	−0.24, 0.18	0.01	−0.21, 0.23	0.26	−0.12, 0.64
**Urban, rural status**								
Rural area Urban area (ref.)	0.07	−0.03, 0.18	0.16	−0.06, 0.37	0.11	−0.12, 0.34	−0.21	−0.64, 0.22
**Urban, rural status × survey wave**								
Rural area × survey wave Urban area × survey wave (ref.)	0.05	−0.10, 0.20	−0.09	−0.40, 0.21	−0.20	−0.54, 0.13	0.18	−0.39, 0.75
**(3) Behavioral/lifestyle model**								
**Inactivity**								
1 h to <2 h 2 h to <4 h 4 h and more Siting <1 h (ref.)	−0.02 −0.07 −0.12	−0.27, 0.22 −0.30, 0.17 −0.36, 0.11	0.05 0.15 −0.001	−0.45, 0.55 −0.33, 0.62 −0.47, 0.47	0.25 0.20 0.27	−0.28, 0.78 −0.30, 0.71 −0.24, 0.77	0.33 0.59 0.58	−0.93, 1.61 −0.63, 1.82 −0.64, 1.80
**Inactivity × survey wave**								
1 h to <2 h × survey wave 2 h to <4 h × survey wave 4 h and more × survey wave Siting <1 h × survey wave (ref.)	0.07 0.20 0.17	−0.36, 0.50 −0.20, 0.61 −0.23, 0.57	−0.03 −0.15 −0.07	−0.93, 0.86 −0.97, 0.68 −0.90, 0.75	0.04 0.12 0.01	−0.94, 1.01 −0.78, 1.03 −0.89, 0.91	−0.03 −0.32 −0.68	−2.03, 1.96 −2.21, 1.57 −2.56, 1.20
**Alcohol consumption**								
14 drinks or less per week 15 drinks or more per week (ref.)	0.04	−0.07, 0.15	−0.04	−0.27, 0.19	−0.04	−0.33, 0.25	0.27	−0.23, 0.78
**Alcohol consumption × survey wave**								
14 drinks or less per week × survey wave 15 drinks or more per week × survey wave (ref.)	0.05	−0.11, 0.20	0.25	−0.05, 0.55	0.05	−0.33, 0.43	−0.57	−1.33, 0.20
**Smoking**								
Not in the last 30 days Smoked (ref.)	0.27^***^	0.12, 0.41	0.57^***^	0.28, 0.85	0.36^*^	0.04, 0.67	0.30	−0.17, 0.77
**Smoking × survey wave**								
Not in the last 30 days × survey wave Smoked × Survey wave (ref.)	0.08	−0.10, 0.27	−0.10	−0.48, 0.27	0.31	0.10, 0.73	0.43	−0.20, 1.06
**Sleep**								
Very satisfied Satisfied Neutral Dissatisfied Very dissatisfied (ref.)	0.93^***^ 0.69^***^ 0.38^***^ 0.25^**^	0.77, 1.09 0.53, 0.84 0.21, 0.55 0.09, 0.41	0.76^***^ 0.59^***^ 0.34 0.12	0.44, 1.08 0.28, 0.90 −0.002, 067 −0.20, 0.44	0.80^***^ 0.61^***^ 0.23 0.24	0.48, 1.13 0.31, 0.91 −0.10, 0.55 −0.07, 0.55	1.27^***^ 0.97^***^ 0.41 0.44	0.73, 1.81 0.49, 1.45 −0.12, 0.95 −0.04, 0.93
**Sleep × survey wave**								
Very satisfied × survey wave Satisfied × survey wave Neutral × survey wave Dissatisfied × survey wave Very dissatisfied × survey wave (ref.)	0.02 0.04 0.16 0.10	−0.19, 0.23 −0.16, 0.25 −0.06, 0.37 −0.12, 0.32	0.14 0.04 0.15 0.13	−0.27, 0.55 −0.35, 0.44 −0.29, 0.58 −0.30, 0.56	−0.01 −0.05 0.30 0.16	−0.43, 0.42 −0.45, 0.35 −0.14, 0.74 −0.27, 0.58	−0.20 −0.17 −0.001 0.18	−0.89, 0.50 −0.85, 0.50 −0.76, 0.76 −0.51, 0.86
**Appetite**								
Very good Good Fair Poor (ref.)	0.40^***^ 0.25^*^ −0.04	0.17, 0.63 0.03, 0.48 −0.29, 0.21	0.46^*^ 0.29 0.07	0.09, 0.83 −0.07, 0.66 −0.34, 0.48	0.38 0.30 0.004	−0.09, 0.85 −0.16, 0.77 −0.51, 0.51	0.26 0.22 −0.22	−0.40, 0.93 −0.43, 0.87 −0.95, 0.51
**Appetite × survey wave**								
Very good × survey wave Good × survey wave Fair × survey wave Poor × survey wave (ref.)	−0.08 −0.21 −0.15	−0.38, 0.22 −0.50, 0.09 −0.49, 0.19	−0.10 −0.19 −0.10	−0.58, 0.39 −0.68, 0.29 −0.67, 0.46	−0.13 −0.27 −0.18	−0.74, 0.49 −0.88, 0.34 −0.86, 0.50	−0.07 −0.30 0.29	−0.92, 0.78 −1.14, 0.55 −0.69, 1.27
**Skipped meals**								
All the time to sometimes Rarely or never (ref.)	−0.31^***^	−0.39, −0.23	−0.18^*^	−0.33, −0.03	−0.33^***^	−0.50, −0.17	−0.20	−0.47, 0.07
**Skipped meals × survey wave**								
All the time to sometimes × survey wave Rarely or never × survey wave (ref.)	0.16^**^	0.07, 0.26	−0.10	−0.30, 0.09	0.16	−0.05, 0.37	0.06	−0.30, 0.42
**(4) Illness context**								
**BMI**								
Underweight Overweight Obese Normal (ref.)	−0.05 0.01 −0.08	−0.39, 0.29 −0.06, 0.09 −0.17, 0.005	1.66^*^ −0.08 −0.16	0.03, 3.29 −0.26, 0.10 −0.35, 0.02	0.05 −0.03 −0.30^**^	−0.54, 0.65 −0.19, 0.14 −0.49, −0.12	−0.28 0.21 −0.06	−0.94, 0.39 −0.10, 0.62 −0.39, 0.28
**BMI × survey wave**								
Underweight × survey wave Overweight × survey wave Obese × survey wave Normal × survey wave (ref.)	−0.13 0.02 −0.13^*^	−0.54, 0.29 −0.07, 0.11 −0.23, −0.03	−2.07^*^ 0.12 −0.004	−4.03, −0.11 −0.10, 0.34 −0.22, 0.21	−0.21 −0.09 −0.14	−0.96, 0.55 −0.30, 0.11 −0.36, 0.08	0.64 −0.13 −0.12	−0.98, 2.25 −0.53, 0.27 −0.53, 0.29
**Self-rated health**								
Excellent Very good Good Fair Poor (ref.)	1.47^***^ 1.21^***^ 0.82^***^ 0.17	1.18, 1.75 0.93, 1.49 0.54, 1.09 −0.11, 0.46	1.41^***^ 1.19^***^ 0.87^***^ 0.26	0.98, 1.84 0.80, 1.59 0.48, 1.26 −0.14, 0.67	1.73^***^ 1.44^***^ 0.96^***^ 0.18	1.21, 2.25 0.94, 1.94 0.47, 1.45 −0.33, 0.68	1.38^**^ 1.40^***^ 0.88* 0.32	0.50, 2.27 0.59, 2.21 0.09, 1.17 −0.49, 1.13
**Self-rated health × survey wave**								
Excellent × survey wave Very good × survey wave Good × survey wave Fair × survey wave Poor × survey wave (ref.)	0.47^*^ 0.37^*^ 0.26 0.25	0.11, 0.83 0.02, 0.72 −0.09, 0.60 −0.11, 0.62	0.64^*^ 0.44 0.23 0.23	0.07, 1.20 −0.07, 0.96 −0.28, 0.74 −0.31, 0.77	0.65 0.56 0.43 0.52	−0.002, 1.30 −0.05, 1.17 −0.17, 1.03 −0.11, 1.16	0.48 0.04 −0.22 −0.24	−0.64, 1.60 −0.94, 1.02 −1.18, 0.73 −1.22, 0.75
**Pain**								
Mild Moderate Severe None (ref.)	−0.15^***^ −0.29^***^ −0.44^***^	−0.23, −0.07 −0.36, −0.21 −0.58, −0.30	−0.21^*^ −0.27^***^ −0.77^***^	−0.38, −0.04 −0.41, −0.13 −1.03, −0.51	−0.08 −0.27^***^ −0.37^**^	−0.26, 0.10 −0.43, −0.12 −0.63, −0.11	−0.14 −0.22 −0.54^*^	−0.48, 0.20 −0.50, 0.06 −0.97, −0.11
**Pain × survey wave**								
Mild × survey wave Moderate × survey wave Severe × survey wave None × survey wave (ref.)	−0.10 −0.10 −0.16	−0.23, −0.07 −0.20, 0.01 −0.37, 0.05	0.10 −0.08 0.20	−0.16, 0.35 −0.28, 0.12 −0.16, 0.57	−0.37^**^ −0.17 −0.39^*^	−0.63, −0.10 −0.39, 0.05 −0.75, −0.04	−0.05 −0.15 0.06	−0.54, 0.45 −0.54, 0.24 −0.57, 0.68
**Number of medicines (baseline)**	−0.03^*^	−0.05, −0.001	−0.05	−0.10, 0.01	−0.08^**^	−0.13, −0.02	−0.11^*^	−0.22, −0.01
**AIC**	40,587.05		10,999.68		9,926.39		3,173.71	

* *p* < 0.05;

** *p* < 0.01;

*** *p* < 0.001;

The reference group in the analysis is indicated by (ref.).

Among the social and environmental factors, number of friends, number of relatives, and housing problems were significantly associated with MRI, but not urban/rural residence. Number of friends had a positive association with MRI (estimate = 0.02, 95% CI: (0.01, 0.02)], and this effect was attenuated between Baseline to FUP1 longitudinally [estimate = −0.01, 95% CI: (−0.01, −0.002)]. Number of relatives was also positively related to MRI score at Baseline [estimate = 0.001, 95% CI: (0.001, 0.002)], and the relationship was strengthened over time [estimate = 0.003, 95% CI: (0.002, 0.004)]. Participants with housing problem(s) reported lower levels of MRI [estimate = −0.31, 95% CI: (−0.39, −0.24)] than those without housing problem(s), although the longitudinal effect was not supported.

Three of the seven behavioral and lifestyle factors were significantly related to MRI scores. Participants who did not smoke in the past 30 days prior to Baseline survey reported a higher MRI score [estimate = 0.27, 95% CI: (0.12, 0.41)]. Sleep was also correlated to the MRI score at Baseline, with higher levels of satisfaction of sleep leading to higher MRI scores [estimate = 0.93, 95% CI: (0.77, 1.09) for very satisfied; estimate = 0.69, 95% CI: (0.53, 0.84) for satisfied; estimate = 0.38, 95% CI: (0.21, 0.55) for very neutral; estimate = 0.25, 95% CI: (0.09, 0.41) for dissatisfied, compared to very dissatisfied]. Also, participants with either very good or good appetite reported higher MRI scores than those with poor appetite at Baseline [estimate = 0.40, 95% CI: (0.17, 0.63) for very good; estimate = 0.25, 95% CI: (0.03, 0.48) for good]. No longitudinal effect of sleep or appetite on MRI score was supported. Skipping meals had both main effect and interactive effect with survey wave on MRI score. Participants who skipped meals sometimes to all the time reported lower MRI scores than those who rarely or never skipped meal [estimate = −0.31, 95% CI: (−0.39, −0.23)], while the difference of MRI scores between these two groups was attenuated over time [estimate = 0.16, 95% CI: (0.07, 0.26)].

All four health contextual factors were significantly associated with MRI scores. The main effect of BMI on the MRI score was not supported, but when compared to participants with normal BMI scores, those who were obese tended to have a greater decease in MRI scores between Baseline and FUP1 [estimate = −0.13, 95% CI: (−0.23, −0.03)]. Participants rated their health as good, very good, and excellent reported significant higher MRI scores than those rated health as poor at Baseline [estimate = 0.82, 95% CI: (0.54, 1.09) for Good; estimate = 1.21, 95% CI: (0.93, 1.49) for Very good; estimate = 1.47, 95% CI: (1.18, 1.75) for Excellent]. In addition, the interactive effect between self-rated health and survey wave was also supported, indicating that participants with very good and excellent levels of health had significantly greater increases in MRI scores than those with poor health over time [estimate = 0.37, 95% CI: (0.02, 0.72) for Very good; estimate = 0.47, 95% CI: (0.11, 0.83) for Excellent]. Pain was also related to MRI in the expected direction, where participants with all three levels of pain reported lower scores in the MRI than participants with no pain at Baseline [estimate = −0.15, 95% CI: (−0.23, −0.07) for Mild; estimate= −0.29, 95% CI: (−0.36, −0.21) for Moderate; estimate = −0.44, 95% CI: (−0.58, −0.30) for Severe]. The number of medications at Baseline was negatively related to MRI scores [estimate = −0.03, 95% CI: (−0.05, −0.001)].

### Cardiovascular/metabolic cluster: Health behavior findings

There were 3,033 participants who identified with two or more Cardiovascular/metabolic illnesses. Given that most of the covariates showed similar patterns with MRI, and our focus is on health behaviors, we only present below the results for the behavioral and lifestyle factors shown in Table 2.

Four out of seven behavioral and lifestyle factors, including smoking status, skipped meals, sleep, and appetite, were significantly related to MRI scores among the Cardiovascular/metabolic cluster. Not smoking was related to higher MRI scores [estimate = 0.57, 95% CI: (0.28, 0.85)], and skipped meal (all the time to sometimes) was associated with lower MRI scores [estimate = −0.18, 95% CI: (−0.33, −0.03)]. Additionally, participants with very satisfied sleep [estimate = 0.76, 95% CI: (0.44, 1.08)] and satisfied sleep [estimate= 0.59, 95% CI: (0.28, 0.90)] reported higher scores in the MRI compared to those with very dissatisfied sleep. Participants with very good appetite were also associated with MRI when compared with those with poor appetite [estimate = 0.46, 95% CI: (0.09, 0.83)].

In addition, participants who were underweight reported higher MRI scores than those who were normal in BMI at Baseline [estimate = 1.66, 95% CI: (0.03, 3.29)]. The significant interactive effect between BMI and survey wave indicated that participants who were underweight had lower MRI scores at FUP1 compared to those who were normal in BMI [estimate = −2.07, 95% CI: (−4.03, −0.11)].

### Musculoskeletal cluster: Health behavior findings

A total of 2,417 participants (65+) belonged to the Musculoskeletal cluster with two or more osteo-related diseases. Table 2 presents the results yielded from the LMM analysis. Among the behavioral and lifestyle factors, the relationship between smoking, satisfaction with sleep, and skipped meals were replicated compared to the Cardiovascular/Metabolic cluster (see Table 2). However, for BMI, only participants who were obese reported lower MRI scores than those who were normal in BMI [estimate = −0.30, 95% CI: (−0.49, −0.12)] at Baseline.

### Mental health cluster: Health behavior findings

There were 753 participants (65+) with two or more mental health conditions. As shown in Table 2, among the behavioral and lifestyle factors, only the satisfaction with sleep was associated with MRI scores, where participants reporting very satisfied sleep [estimate = 1.27, 95% CI: (0.73, 1.81)] and satisfied sleep [estimate = 0.97, 95% CI: (0.49, 1.45)] reported higher MRI scores than those with very dissatisfied sleep at Baseline. See Table 2 for full results, including all covariates.

## Discussion

A growing body of resilience and aging literature is developing in numerous sub-fields within gerontology multimorbidity (25, 27, 30, 38, 41, 42, 44). This paper is the first to employ longitudinal data separated by ~3 years (2011–2015, and 2015–2018) to investigate the association between a comprehensive set of modifiable health behaviors and multimorbidity resilience (MR) among older adults. Our results provide evidence that behavioral lifestyle factors may be modified to act as resources for those experiencing the multimorbidity processes to enhance resilience.

Among older adults with multimorbidity, higher scores on the MRI were associated with not smoking, higher satisfaction with sleep, better appetite, and fewer skipped meals. In addition, being obese decreased multimorbidity resilience over time, although an association at baseline was not observed. Further, the association for skipped meals with MRI was attenuated between the survey time periods. This research adds to prior research in several unique ways by demonstrating that health behaviors play an important role in the ways in which older adults adapt to multimorbidity resilience *over time*, which is often missing in research studies on resilience and aging processes.

Our results on smoking status parallel a large body of research showing that smoking increases multimorbidity risk [for example, (38, 42, 44, 75, 76)]. Moreover, this study also establishes that not smoking fosters resilience fortitude, which is consistent with an earlier study using the MRI but based on cross-sectional data (30). Looking at the risk side of the equation, smoking compromises MR due to its addictive properties among smokers coupled with its adverse effect on quality of life and psychological wellbeing (77). The importance of sleep quality as a positive health behavior for MR underscores its influence for illness recovery as well as role and identity reintegration, and is consistent with research demonstrating the salience of good sleep for better general resilience and lower health care utilization (30, 46, 78). Additionally, having a good appetite and not skipping meals appear to enhance levels of resilience among those with multimorbidity and is consistent with a growing number of studies showing the importance of food security in patterns of multimorbidity (76, 79), as well as resilience (30). Yet, the association between obesity and MR was only found when examining this relationship longitudinally. This is consistent with past research that has shown an increased risk of multimorbidity associated with obesity over time (42, 76). Taken together, our results on multimorbidity resilience are consistent with the broader research focusing on predictors of multimorbidity, which can provide a useful benchmark upon which we can validate the findings on resilience. For example, Skivington et al. (76) found support for similar behavioral lifestyle factors on multimorbidity (2+ conditions) in a Scottish longitudinal study. After controlling for socio-demographic covariates, multimorbidity risk was higher among smokers compared to non-smokers (OR 1.38, 95% CI 1.20–1.60); for those with BMI 30–35 (OR 1.57, 95% CI 1.22–2.01) and >35 (OR 2.21, 95% CI 1.40–3.48) compared to BMI 20–25; and for those with poor diet (OR 1.28, 95% CI 1.05–1.57). Although BMI is considered to be a health factor, we consider it to be an indicator of eating habits and physical activity level, similar to other studies [e.g., (30, 42, 80)].

However, our study did not support an association between physical inactivity and multimorbidity resilience. Previous research pertaining to the influence of physical inactivity on multimorbidity has been equivocal. Studies have reported positive associations among older adults with multimorbidity (75), support only for older men in others (41), and non-support in others, including longitudinal studies [e.g., (42, 45, 76)]. The inconsistent findings for physical inactivity may be the result of difference in design, age of the target population, and measurement, suggesting the need for more studies. It is also possible that physical activity, rather than sedentary time, may bolster multimorbidity resilience, since each exert independent effects on health (81, 82). Thus, physical activity has multipotent effects on physical function and mental health (83) that may improve resilience. Additionally, the absence of support for drinking patterns as a predictor of MR is aligned to many other findings based on research in the health and aging literature, some of which uses cross-sectional designs that can convolute the direction of the association since many individuals with multimorbidity reduce drinking, which does not mean that drinking is a protective factor [e.g., (39)].

Supplementary analyses were conducted on the associations of all health behaviors and covariates for the three multimorbidity clusters: Cardiovascular/metabolic, Musculoskeletal and Mental health. For the two physical multimorbidity clusters (Cardiovascular/metabolic, and Musculoskeletal), most of the findings reported for the 2+ multimorbidity group were replicated (see Table 2). The exception is the Mental-health cluster, where only sleep satisfaction was found to be associated with MR. Another notable exception was that obesity related to MRI in the musculoskeletal cluster only, which may reflect the additional loading demands of obesity on the musculoskeletal system (84). Additionally, being underweight related to lower MRI in the cardiovascular/metabolic disease cluster only, both at baseline and over time. Underweight BMI has previously been associated with increased mortality in Canadian seniors (85). It is possible that trajectories of body weight changes may have disease-specific impacts on health outcomes and resiliency. However, these results should be interpreted with caution.

This study also identified relationships between several socio-demographic resources and health care factors and multimorbidity resilience. Among the full 2+ multimorbidity group, increased resilience scores were observed among those with lower age, being male compared to female, having post-secondary education, higher income levels, being married or partnered, having larger support networks, and fewer housing problems. These social determinants are consistent with studies on multimorbidity as well as formative resilience research among older adults (6, 14, 25, 30, 36, 38, 42, 76). Turning to the health context measures, higher multimorbidity resilience was associated with better perceived health, less perceived pain, and fewer medications. These findings concur with other research on both multimorbidity and resilience (10, 21, 30, 53).

A number of limitations of these analyses are notable. First, the measures incorporated in the analyses are restricted to those available in the CLSA data sets. Additional modifiable health behaviors (e.g., meditation, sexuality, etc.) may also influence MR. Second, the MR index is a new measure that has only been validated in one study and used in a small cluster of studies to date, thus requiring further analyses and comparisons to other resilience measures (5, 10, 30). For instance, established measures, such as the Connor-Davidson Resilience Score (86), or the Brief Resilient Coping Scale (87), or a priori statistical methods of estimating resilience (16), could be employed to further validate these findings and advance the literature surrounding multimorbidity resilience. Third, given that multimorbidity is variable due to differing symptom presentation and illness severity (e.g., hypertension, cancer, diabetes, etc.), research that incorporates additional illness context factors, such as onset, severity, and duration, and examines interactions with health behavior may help to specify the treatable, or modifiable moments in illness trajectories (88). Fourth, research may benefit by examining the cumulative effects of modifiable health behaviors over time, which has been useful in the broader multimorbidity risk literature (44, 75). This could include combinations of not smoking, physical activity, maintaining positive eating habits and a healthy weight, and quality sleep. Finally, this work needs to be extended to additional sub-groups, such as diverse racial/ethnic groups (89), those without health care insurance (6, 33), and at a community or system level (25, 28), especially during the COVID-19 pandemic (8, 17, 90, 91).

## Conclusion

The ability to adapt, bounce back or reintegrate from multiple chronic illnesses, termed multimorbidity resilience, is fundamental to healthy aging and is receiving increasing attention in the literature (2, 6, 11, 12, 14, 26, 27, 30, 31, 91), including pandemic research (88, 90). Our findings indicate that there are several mutable health behaviors that are associated with MR and worthy of considering for intervention. The health behaviors found to be important in this study can be used to tailor and target health promotion and public health programs and policies. Innovations in the delivery of interventions for older adults with multimorbidity may utilize these findings to develop and implement innovative health promotion approaches (e.g., multifactor telehealth counseling, digital behavioral monitoring devices, community support programs, peer support groups, tailored cognitive therapy, etc.). Indeed, proactive, strength-based approaches to enhance resilience may prove to be valuable in enhancing aging well. Finally, several known social determinants of multimorbidity also have been found to be important, including age, gender, socio-economic deprivation factors and social support, which may also be low hanging fruit in the development of interventions targeting MR. The present study serves to advance important findings for other studies to build upon regarding the complex ways in which resilience can be elucidated and enhanced among persons experiencing multimorbidity over the life course.

## Data availability statement

Publicly available datasets were analyzed in this study. This data can be found here: https://www.clsa-elcv.ca/data-access.

## Ethics statement

This current project received ethics approval at two levels. Consent to participate was obtained for all participants under the CLSA harmonized multi-university ethics process approved by the Hamilton Integrated Research Ethics Board (HiREB), Hamilton Health Sciences/McMaster University. Written consent was obtained from all CLSA participants prior to enrollment. Individuals who were not deemed to be cognitively functional were excluded from the CLSA study. Simon Fraser University (SFU) was a participating institution in the CLSA data collection, and the SFU Office of Research Services Ethics Committee reviewed all consent material prior to data collection (SFU ORS #2010s0281). The patients/participants provided their written informed consent to participate in this study.

## Author contributions

AW wrote the manuscript. LL conducted analyses of data, drafted methods, and results sections. CW, JF, KK, and IL reviewed and edited the manuscript. All authors have read and approved the final version of the manuscript, and have agreed to be accountable for all parts.

## Funding

This research was made possible using the data/biospecimens collected by the Canadian Longitudinal Study on Aging (CLSA). Funding for the Canadian Longitudinal Study on Aging (CLSA) is provided by the Government of Canada through the Canadian Institutes of Health Research (CIHR) under grant reference: LSA 94473 and the Canada Foundation for Innovation, as well as the following provinces, Newfoundland, Nova Scotia, Quebec, Ontario, Manitoba, Alberta, and British Columbia. This research has been conducted using the CLSA Comprehensive Cohort dataset [version 1.0], under Application Number [#150914] (https://www.clsa-elcv.ca/). The CLSA is led by Drs. Parminder Raina, Christina Wolfson and Susan Kirkland.

## Conflict of interest

The authors declare that the research was conducted in the absence of any commercial or financial relationships that could be construed as a potential conflict of interest.

## Publisher's note

All claims expressed in this article are solely those of the authors and do not necessarily represent those of their affiliated organizations, or those of the publisher, the editors and the reviewers. Any product that may be evaluated in this article, or claim that may be made by its manufacturer, is not guaranteed or endorsed by the publisher.
